# HIF1α overexpression enhances diabetic wound closure in high glucose and low oxygen conditions by promoting adipose-derived stem cell paracrine function and survival

**DOI:** 10.1186/s13287-020-01654-2

**Published:** 2020-04-05

**Authors:** Jin Xu, Xiaoyu Liu, Feng Zhao, Ying Zhang, Zhe Wang

**Affiliations:** 1 Department of Surgery, Shengjing Hospital of China Medical University, No. 36 Sanhao Street, Heping District, Shenyang, 110004 China; 2 Department of Obstetrics and Gynecology, Reproductive Medicine Center, Shengjing Hospital of China Medical University, No. 36 Sanhao Street, Heping District, Shenyang, 110004 China; 3 Department of Stem Cells and Regenerative Medicine, Shenyang Key Laboratory for Stem Cells and Regenerative Medicine, Key Laboratory of Cell Biology, Ministry of Public Health, and Key Laboratory of Medical Cell Biology, Ministry of Education, China Medical University, No. 77 Puhe Street, Shenbei New District, Shenyang, 110122 China; 4 Department of Pathology, Shengjing Hospital of China Medical University, No. 36 Sanhao Street, Heping District, Shenyang, 110004 China

**Keywords:** Hypoxia-inducible factor 1α, Adipose-derived stem cells, Wound healing, High glucose, Reactive oxygen species, Mice, Stem cell transplantation

## Abstract

**Background:**

Adipose-derived stem cell (ADSC) transplantation is a promising strategy to promote wound healing because of the paracrine function of stem cells. However, glucose-associated effects on stem cell paracrine function and survival contribute to impaired wound closure in patients with diabetes, limiting the efficacy of ADSC transplantation. Hypoxia-inducible factor (HIF)1α plays important roles in wound healing, and in this study, we investigated the effects of HIF1α overexpression on ADSCs in high glucose and low oxygen conditions.

**Methods:**

Adipose samples were obtained from BALB/C mice, and ADSCs were cultured in vitro by digestion. Control and HIF1α-overexpressing ADSCs were induced by transduction. The mRNA and protein levels of angiogenic growth factors in control and HIF1α-overexpressing ADSCs under high glucose and low oxygen conditions were analyzed by quantitative reverse transcription-polymerase chain reaction and western blotting. The effects of ADSC HIF1α overexpression on the proliferation and migration of mouse aortic endothelial cells (MAECs) under high glucose were evaluated using an in vitro coculture model. Intracellular reactive oxygen species (ROS) and 8-hydroxydeoxyguanosine (8-OHdG) levels in ADSCs were observed using 2,7-dichlorodihydrofluorescein diacetate staining and enzyme-linked immunosorbent assays, respectively. Apoptosis and cell cycle analysis assays were performed by flow cytometry. An in vivo full-thickness skin defect mouse model was used to evaluate the effects of transplanted ADSCs on diabetic wound closure.

**Results:**

In vitro, HIF1α overexpression in ADSCs significantly increased the expression of vascular endothelial growth factor A, fibroblast growth factor 2, and C-X-C motif chemokine ligand 12, which were inhibited by high glucose. HIF1α overexpression in ADSCs alleviated high glucose-induced defects in MAEC proliferation and migration and significantly suppressed ADSC ROS and 8-OHdG levels, thereby decreasing apoptosis and enhancing survival. In vivo, HIF1α overexpression in ADSCs prior to transplantation significantly enhanced angiogenic growth factor expression, promoting wound closure in diabetic mice.

**Conclusions:**

HIF1α overexpression in ADSCs efficiently alleviates high glucose-induced paracrine dysfunction, decreases oxidative stress and subsequent DNA damage, improves viability, and enhances the therapeutic effects of ADSCs on diabetic wound healing.

## Background

Diabetes mellitus is a serious chronic metabolic disorder characterized by high glucose levels, which induces oxidative stress. It affects more than 400 million people worldwide, with a predicted > 50% increase in cases by 2030 [1–3]. Micro- and macrovascular diseases are key complications of diabetes and may be important risk factors for death in diabetic patients [4, 5]. Endothelial dysfunction is strongly associated with the risk of vascular disease and results in impaired wound healing [5–7]. Previous studies have shown that a high glucose concentration downregulates the expression of various angiogenic growth factors, such as vascular endothelial growth factor A (VEGFA), fibroblast growth factor 2 (FGF2), and C-X-C motif chemokine ligand 12 (CXCL12), which in turn suppress endothelial cell (EC) functions, leading to impaired angiogenesis and delayed wound closure [8].

Adipose-derived stem cells (ADSCs) support the engineering of functional tissue by secreting angiogenic and cytoprotective factors, which act in a paracrine fashion to promote vascularization and cell survival [9]. Due to this paracrine function, ADSCs are being increasingly investigated as cellular therapies for different diseases, as well as for burns and trauma. However, diabetes impairs the paracrine function and survival of transplanted stem cells [10]. Previous in vitro studies have shown that high glucose induces reactive oxygen species (ROS) production and subsequent ADSC apoptosis [11]. Therefore, an effective method to improve stem cell paracrine function and survival in the diabetic microenvironment is urgently needed [12].

Hypoxia-inducible factor 1 (HIF1) is a master regulator of oxygen homeostasis and an important determinant of healing outcomes. HIF1 contributes to all stages of wound healing through its roles in migration, survival under hypoxia, cell division, growth factor release, and matrix synthesis. HIF1α is the regulatory subunit of HIF1 [13]. Positive regulators of HIF1α, such as prolyl-4-hydroxylase inhibitors, enhance diabetic ischemic wound closure and are currently undergoing clinical trials for the treatment of several human ischemic conditions [14]. Specifically, HIF1α enhances *VEGFA* expression by directly binding its promoter [15]. However, the protective effects of ADSC HIF1α on diabetic wound closure have not been reported.

In this study, we examined whether HIF1α overexpression in ADSCs improves diabetic wound closure in mice and investigated the possible mechanisms involved. These data reveal an effective strategy to enhance wound healing treatment under diabetic conditions.

## Materials and methods

### Ethical considerations

This study was approved by the Laboratory Animal Welfare and Ethics Committee of China Medical University and conducted according to the Guide for the Care and Use of Laboratory Animals.

### Isolation and treatment of mouse ADSCs

Twenty BALB/c mice (male; body weight 18–24 g; 8 weeks old) were purchased from Beijing Huafukang Bioscience Co. Inc. (Beijing, China). ADSCs were isolated from the inguinal fat pad as previously described [16]. Cells were suspended in Dulbecco’s modified Eagle’s medium (DMEM) supplemented with 10% fetal bovine serum, 100 U/mL penicillin, and 100 μg/mL streptomycin. The cultures were maintained at 37 °C in a humidified atmosphere containing 5% CO_2_. ADSCs were used in experiments after 3–6 passages. To overexpress HIF1α, ADSCs were transduced with either an empty lentivirus (as a control) or a lentivirus expressing recombinant HIF1α using a cytomegalovirus promoter (Origene, Shanghai, China) for 48 h. For high glucose experiments, cells were cultured in serum-free DMEM containing high glucose (25 mM) for 24 h prior to lentiviral transduction. Cells cultured with 5.5 mM glucose were used as normoglycemic controls. After 48 h, cells were harvested to detect mRNA and protein expression. All experiments were performed in a hypoxia chamber (Mitsubishi GAS Chemical, Tokyo, Japan; oxygen concentration: 0.1%) as previously described [17]. For ADSC transplantation experiments, donor ADSCs transduced with empty or HIF1α-encoding lentiviruses were subcutaneously injected at the time of skin wound induction.

### Quantitative reverse transcription-polymerase chain reaction (RT-qPCR)

VEGFA, HIF1α, FGF2, and CXCL12 mRNA levels in ADSCs or tissues were examined by RT-qPCR as previously reported [17]. PCR primer sequences are listed in Table 1. CXCL12 primers (QT00161112) were purchased from Qiagen (Valencia, CA, USA). Briefly, after reverse transcription, qPCR was performed using the iQ5 real-time PCR detection system (Bio-Rad, Hercules, CA, USA) with SYBR Supermix (Qiagen). The fold change in the expression levels of each target mRNA under experimental and control conditions was calculated using the 2^−ΔΔCT^ method [18], relative to glyceraldehyde 3-phosphate dehydrogenase (GAPDH).

**Table 1:** Primers used in RT-qPCR analysis

Transcript	Primer (5′-3′)	Product length
HIF1α	Forward: CAAGAAACCACCCATGAC Reverse: GGCTCATAACCCATCAAC	165 bp
VEGFA	Forward: AGGGCAGAATCATCACGAAGT Reverse: AGGGTCTCGATTGGATGGCA	75 bp
FGF2	Forward: GGGACTGGTCAGTATTAGAGGT Reverse: CTCTTGGAGTTCCGTCTTTGTT	215 bp
GAPDH	Forward: CTGCCCAGAACATCATCC Reverse: CAGATGCCTGCTTCAC	197 bp

### Western blotting

VEGFA, HIF1α, FGF2, and CXCL12 protein levels in ADSCs and tissues were determined by western blotting as previously described [11, 12]. Briefly, after electrophoresis and transfer to polyvinylidene difluoride membranes, proteins were incubated with antibodies against HIF1α (ab216842, 1:500, Abcam, Cambridge, MA, USA), anti-VEGFA (ab1316, 1:100, Abcam), FGF2 (ab92337; 1:300; Abcam), CXCL12 (ab137867; 1:300; Abcam), and GAPDH (#5174; 1:20,000; Cell Signaling Technologies, Danvers, MA, USA) at 4 °C overnight. Proteins were visualized using an enhanced chemiluminescence detection system (Bio-Rad, Hercules, CA, USA) and exposure to X-ray film and quantified by laser scanning densitometry (GE Healthcare Life Sciences, Piscataway, NJ). GAPDH was used as a loading control.

### ADSC-MAEC coculture

MAECs were purchased from CHI Scientific (Maynard, MA, USA) and cultured in endothelial basal medium (CHI Scientific) at 37 °C in a humidified incubator with 95% O_2_ and 5% CO_2_. To examine the effects of ADSCs on MAEC proliferation and migration, MAECs were seeded at 0.5 × 10^4^ cells/cm^2^ in 24-well plates and grown to 80–90% confluence, then grown in serum-free medium for 12 h prior to coculture. For high glucose experiments, control or HIF1α-overexpressing ADSCs were seeded at 0.5 × 10^4^ cells/cm^2^ in CoStar Transwell cell culture inserts (0.4 μm pore size; Corning, Corning, NY, USA) and cultured in serum-free DMEM containing high glucose (25 mM) for 24 h prior to coculture. For vascular endothelial growth factor receptor 2 (VEGFR2) inhibitor (ZD6474) experiments, MAECs were pre-treated with ZD6474 for 24 h prior to coculture with ADSCs. In group 1 (High glucose), control ADSCs were cocultured with MAECs; in group 2 (High+HIF1α), HIF1α-overexpressing ADSCs were cocultured with MAECs; in group 3 (High+HIF1α + ZD6474), HIF1α-overexpressing ADSCs were cocultured with MAECs pre-treated with ZD6474; and in group 4 (High+ZD6474), control ADSCs were cocultured with MAECs pre-treated with ZD6474. For MAEC proliferation and apoptosis assays, MAECs were harvested after 2 and 3 d of coculture with ADSCs, respectively.

### Cell proliferation assays

The 3-(4, 5-dimethylthiazol-2-yl)-2, 5-diphenyltetrazolium bromide (MTT) assay (Andwin Scientific, Addison, IL, USA) was used to assess relative cell growth and viability according to the manufacturer’s instructions. ADSCs or MAECs (5 × 10^3^) were seeded into 96-well culture plates for 24 h before incubation with MTT for 4 h at 37 °C. The absorbance of the medium was measured at 540 nm, and the data are expressed as ratios of the control value. Three independent experiments were conducted, with two technical replicates per experiment.

### Apoptosis assays

Apoptotic cells were quantified using the Annexin V-FITC/PI Apoptosis Detection Kit (KeyGen Biotech, Jiangsu, China) according to the manufacturer’s instructions. Briefly, cells were trypsinized and resuspended in binding buffer containing Annexin V-fluorescein isothiocyanate (FITC) and propidium iodide (PI) at 22–24 °C for 10 min before analysis on a FACSCalibur flow cytometer (BD Biosciences, San Jose, CA). Three independent experiments were conducted, with two technical replicates per experiment.

### Transwell migration assays

MAEC migration was measured using Transwell migration assays (8.0 μm pore size; Corning-Costar) according to the manufacturer’s protocol. In brief, ADSCs were seeded in the bottom chamber of 24-well plates at 0.5 × 10^4^ cells/cm^2^. A cell culture insert with 8.0 μm pores was placed in the well, and MAECs were added at 0.5 × 10^4^ cells/cm^2^ and incubated at 37 °C in 5% CO_2_ for 24 h. Following incubation, the filters were removed and cells remaining on the upper surface of the membrane were removed with a cotton swab. The membranes were washed with PBS two times, and cells adhering beneath the membrane were fixed in 4% paraformaldehyde and stained with 4′,6-diamidino-2-phenylindole [19]. The number of migrated cells was quantified by counting eight randomly selected regions under a fluorescence microscope (Leica Wetzlar, Germany).

### In vitro wound healing assays

We also used scratch assays to estimate the migration ability of MAECs in vitro. Briefly, MAECs were seeded in the bottom chamber of 24-well plates at 0.5 × 10^4^ cells/cm^2^ and grown to 80–90% confluence, then grown in serum-free medium for 12 h. Then, the confluent layer of cells was scratched using a sterile 200 μL pipette tip. After washing with PBS, MAECs were cocultured with ADSCs. Images were recorded immediately after the monolayers were scratched (0 h) and 24 h later. Scratched areas were measured using Image-Pro Plus 6.0 software. The results are presented as the percentage of wound closure, which was calculated as follows: [wound area (initial) − wound area(final)]/wound area (initial) × 100 [10, 15]. Three independent experiments were conducted, with two technical replicates per experiment.

### ROS measurements

Intracellular ROS levels were assessed using the peroxide-sensitive fluorescent probe 2,7-dichlorodihydrofluorescein diacetate (DCFH-DA, Beyotime Institute of Technology, Jiangsu, China) [20]. ADSCs were cultured as described and subsequently exposed to 10 mol/L DCFH-DA for 30 min at 37 °C. Fluorescence intensity was observed in a DMI6000B inverted microscope (Leica, Wetzlar, Germany). Three independent experiments were conducted, with two technical replicates per experiment.

### Enzyme-linked immunosorbent assays (ELISAs)

To detect DNA damage, 8-hydroxydeoxyguanosine (8-OHdG) was detected using the 8-OHdG ELISA kit (BYE10099; Shanghai Bangyi Biotechnology, Shanghai, China) according to the manufacturer’s instructions. ADSCs were washed twice with PBS, lysed in 100 μL radioimmunoprecipitation buffer (Beyotime Institute of Technology), and centrifuged at 3000×*g* for 20 min at 4 °C. Each sample (10 μL) was combined with 40 μL sample diluent and added to the enzyme-labeled plate before sealing and incubation at 37 °C for 30 min. After incubation, the absorbance was measured at 450 nm. Three independent experiments were conducted, with two technical replicates per experiment.

### Cell cycle analysis

ADSCs were detached from plates, centrifuged, and resuspended, washed with PBS, and fixed in 70% ethanol at 4 °C for 24 h. Cells were then suspended in 300 μL dyeing solution (500 μL buffer containing 10 μL RNase A and 25 μL PI) and incubated at 37 °C for 30 min in the dark. Cells (1 × 10^4^) were subjected to cell cycle analysis on a FACSCalibur flow cytometer (BD Biosciences). Data are from three independent experiments were conducted, with two technical replicates per experiment.

### ADSC labelling

To track ADSCs in vivo, control- and HIF1α-transduced ADSCs were grown to confluence in cell culture dishes and on glass slides and treated with 20–30 μg/mL superparamagnetic polyethyleneimine-coated iron oxide nanoparticles (SPIONs) (Nanjing Nanoeast Biotech, Nanjing, China) for 24 h [21].

### Diabetic wound closure model

To evaluate the therapeutic effects of HIF1α-overexpressing ADSCs on diabetic wound closure in vivo, a mouse model was established. Briefly, following fur removal from the dorsal surface, the mice were anesthetized with sodium pentobarbital (0.5 mg/g), and a 10-mm full-thickness round excisional skin wound was made on the back of each mouse with a sterile 10-mm punch biopsy tool. BALB/c mice (male; body weight 18–24 g; 8 weeks old) were randomly divided into three groups (*n* = 12 mice/group). In all three groups, diabetes was induced by intraperitoneal (i.p.) injection of 150 mg/kg body weight streptozotocin (STZ). Mice with blood glucose levels ≥ 16.7 mmol/L were considered diabetic. One week later, 36 wounds were generated. Group 1 (high glucose) mice were left untreated, group 2 (high+ADSCs) mice received control ADSCs, and group 3 (high+ADSCs-HIF1α) mice received HIF1α-overexpressing ADSCs. ADSCs were incubated with SPIONs for in vivo cell tracking using Prussian blue staining. Control and HIF1α-overexpressing ADSCs (1 × 10^6^ in 60 μL PBS) were subcutaneously injected with a 25-gauge needle at the time of skin wound induction, and wounds were monitored for 2 weeks.

### Statistical analysis

Data from repeated experiments are presented as the mean ± standard deviation (SD). Data were compared between groups using one- or two-way analysis of variance (ANOVA). Following ANOVA, the least significant difference post hoc test or Dunnett’s *t* test with Bonferroni correction was used to analyze the significance of differences between the mean values of the experimental and control groups. *P* < 0.05 was considered significant.

## Results

### HIF1α overexpression promotes ADSC paracrine function

**Fig. 1:**
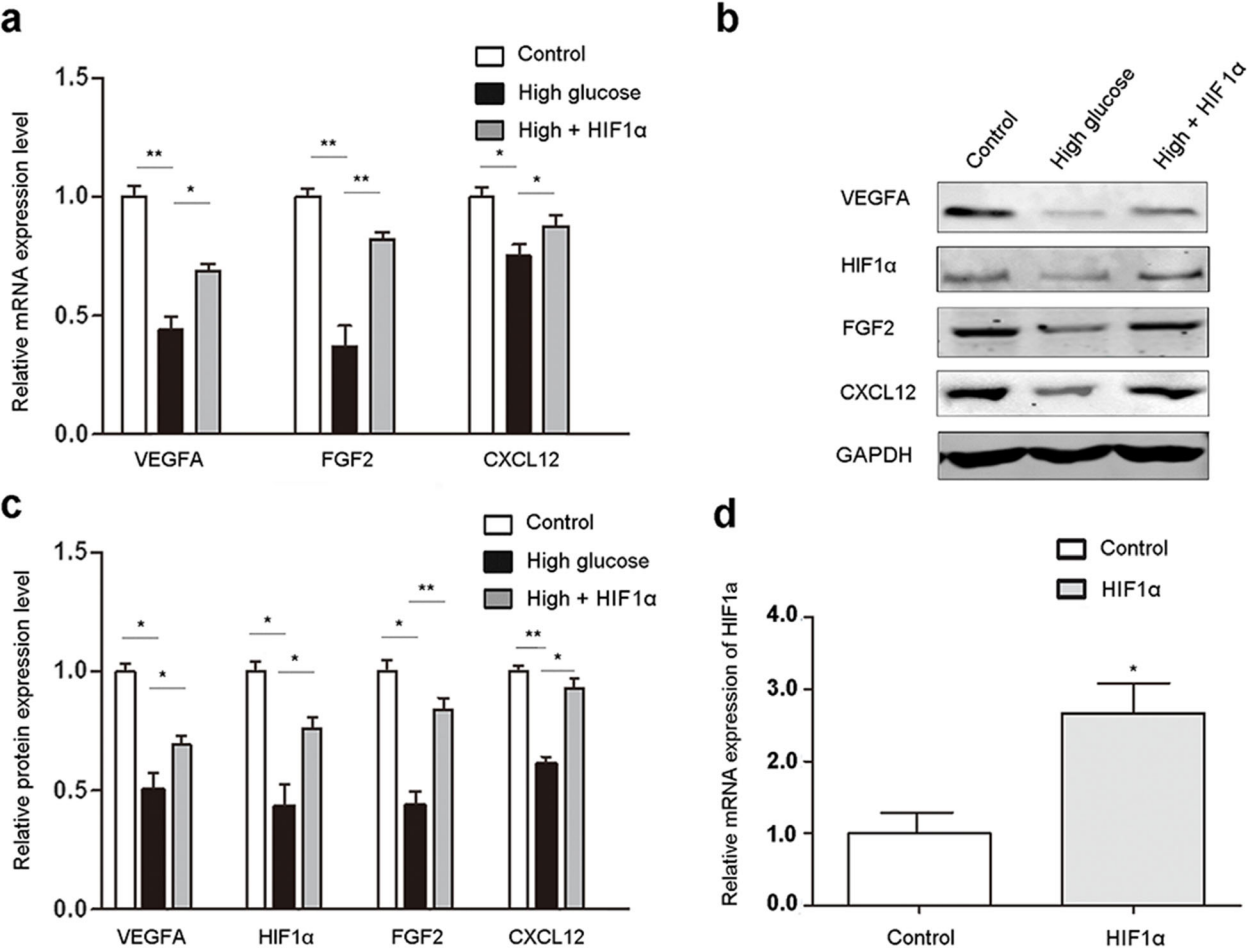
HIF1α overexpression in ADSCs significantly increases angiogenic growth factor production in high glucose/low oxygen. VEGFA, FGF2, and CXCL12 expression levels in HIF1α-overexpressing ADSCs under high glucose and low oxygen. **a** mRNA expression levels were analyzed by RT-qPCR. **b** Protein expression levels were analyzed by western blotting and **c** quantified. **d** HIF1α mRNA levels in transduced ADSCs were determined by RT-qPCR. Cells cultured in normal glucose levels and treated with PBS were used as controls. GAPDH was used for normalization in RT-qPCR experiments and as a loading control in western blots. **p < 0.01; *p < 0.05; Control, normoglycemia; High, high glucose; HIF1α, HIF1α overexpression

To evaluate the effects of HIF1α on diabetic wound closure, we isolated ADSCs and transduced them with control or lentiviruses expressing either null (as a control) or recombinant HIF1α under a CMV promoter. HIF1α mRNA levels in the transduced cells were determined by RT-qPCR (Fig. 1a). While high glucose and low oxygen disrupted VEGFA, FGF2, and CXCL12 expression, HIF1α overexpression in ADSCs restored their expression at both the mRNA (Fig. 1b) and protein (Fig. 1c, d) levels. FGF2 and CXCL12 promote cell migration, while VEGFA promotes cell survival during oxidative stress. These results demonstrate that HIF1α overexpression in ADSCs enhances their paracrine function in high glucose/low oxygen conditions.

### HIF1α overexpression in ADSCs alleviates high glucose-induced MAEC dysfunction

**Fig. 2:**
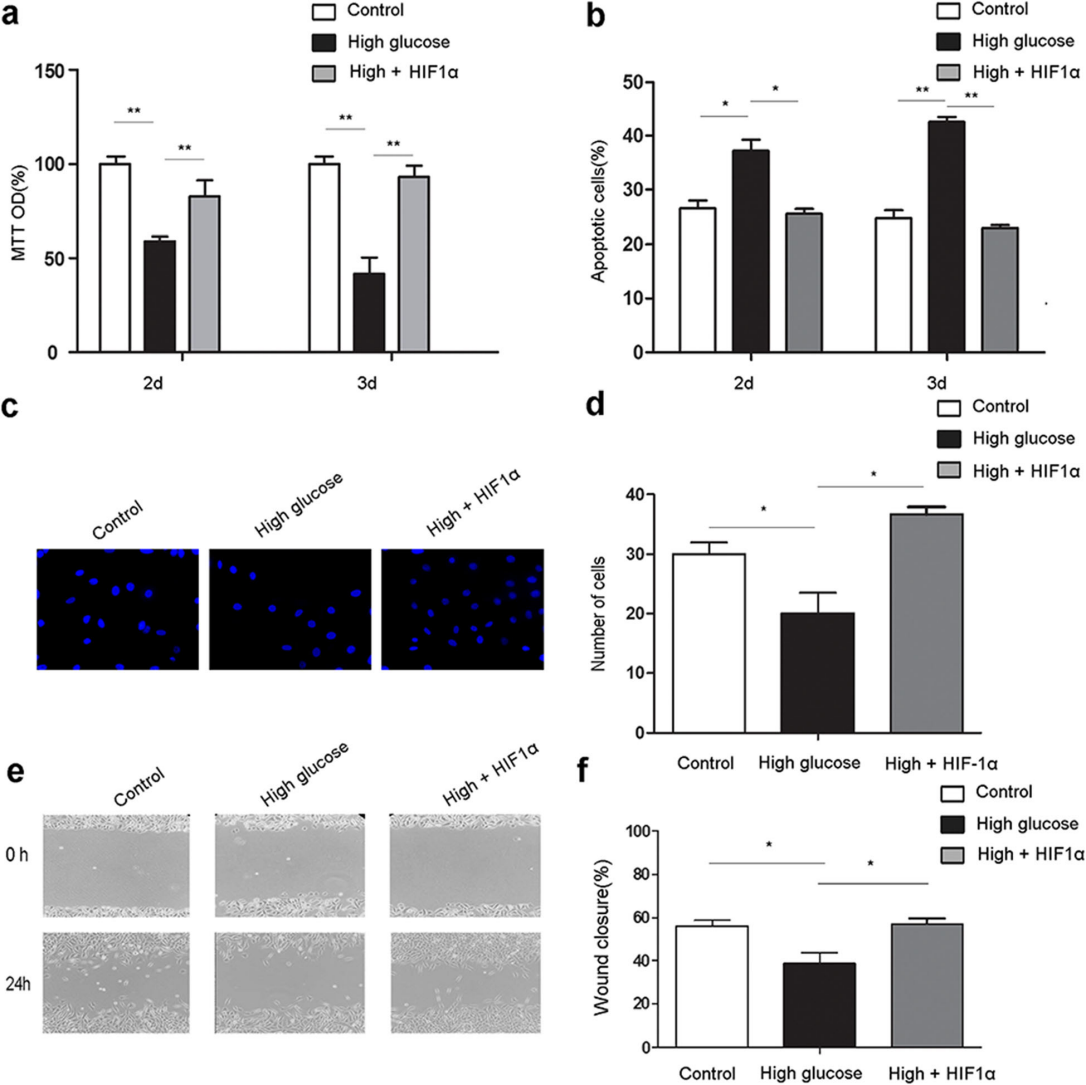
HIF1α overexpression in ADSCs alleviates MAEC dysfunction in high glucose/low oxygen conditions. **a** The proliferation of MAECs cocultured with control or HIF1α-overexpressing ADSCs was analyzed by MTT assay. **b** After coculture with control or HIF1α-overexpressing ADSCs under high glucose, apoptotic MAECs were detected. **c** Representative images from MAEC migration assays at 24 h. **d** Quantitation of MAEC in vitro migration assays. **e** Representative images from MAEC wound healing assays at 0 and 24 h. **f** Quantitation of MAEC in vitro wound healing assays. All experiments were performed under hypoxia. Cells cultured in normal glucose levels and treated with PBS were used as controls. **p < 0.01; *p < 0.05; Control, normoglycemia; High, high glucose; HIF1α, HIF1α overexpression

EC dysfunction under high glucose has been shown to impair wound healing [7]. Therefore, we established a coculture model to explore the effects of HIF1α-overexpressing ADSCs on MAEC function in a high glucose environment. Proliferation and migration are crucial EC functions, and impairment of these processes can prevent angiogenesis in the wound area and delay wound healing [7, 22]. In MAECs, high glucose decreased proliferation (Fig. 2a), increased apoptosis (Fig. 2b), and decreased migration (Fig. 2c–f), while HIF1α-overexpressing ADSCs restored these functions.

**Fig. 3:**
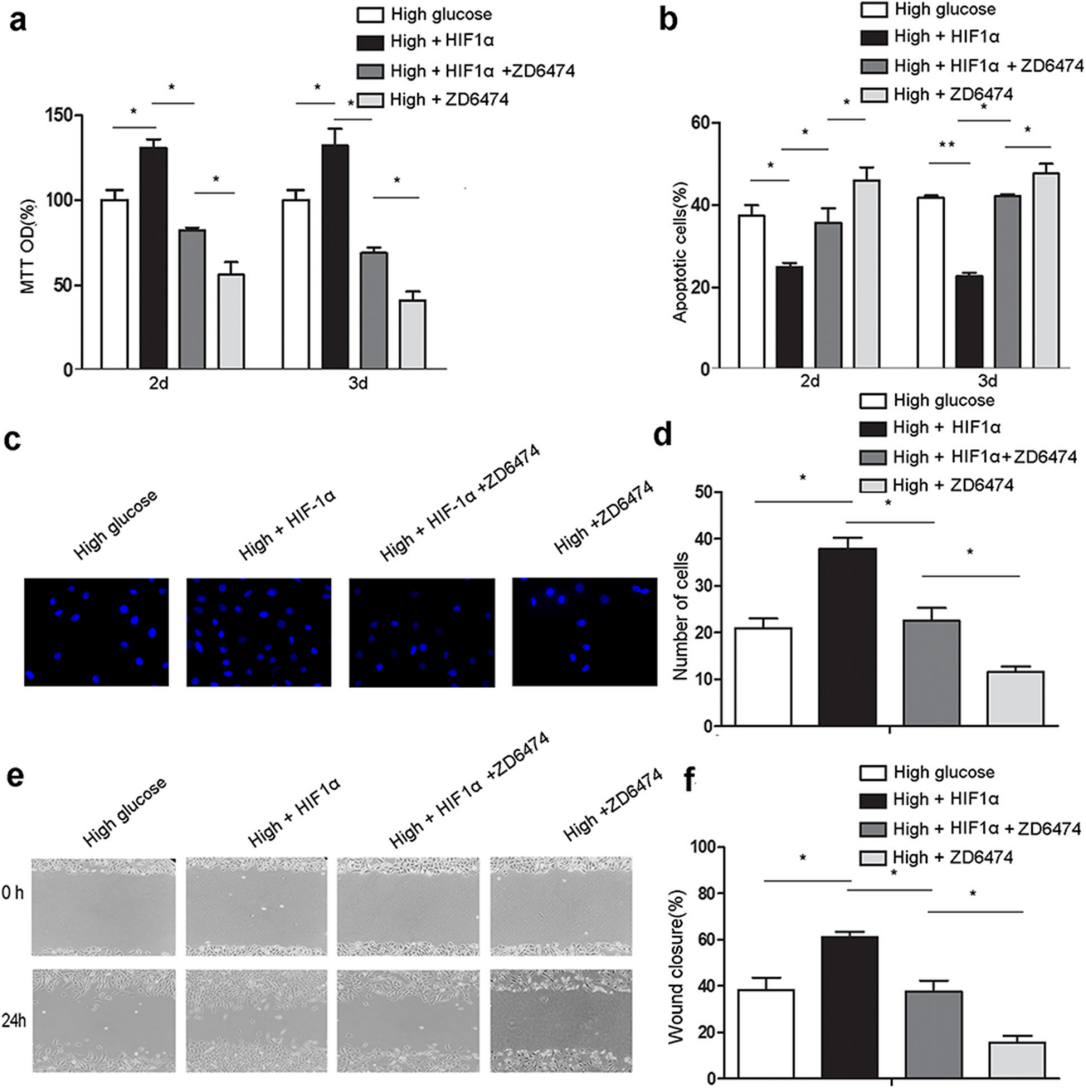
VEGFR2 mediates HIF1α-overexpressing ADSC-induced protection of MAECs in high glucose/low oxygen. **a** The proliferation of MAECs treated with the VEGFR2 inhibitor ZD6474 and cocultured with control or HIF1α-overexpressing ADSCs under high glucose was analyzed by MTT assay. **b** Apoptotic MAECs after coculture as in **a**. **c** Representative images of MAEC wound healing assays at 0, 24, and 48 h (scale bars, 200 μm). **d** MAEC wound healing after 0, 24, and 48 h. All experiments were performed under hypoxia. Cells cultured in normal glucose levels and treated with PBS were used as controls. **p < 0.01; *p < 0.05; High, high glucose; HIF1α, HIF1α overexpression; ZD6474, cells treated with ZD6474

We next investigated whether HIF1α overexpression in ADSCs alleviates high glucose-induced MAEC dysfunction by regulating VEGFR2 expression. We analyzed the proliferation, apoptosis, and migration of MAECs treated with both HIF1α overexpressing-ADSCs and a VEGFR2 inhibitor (ZD6474) in high glucose. ZD6474 treatment decreased proliferation (Fig. 3a), increased apoptosis (Fig. 3b), and decreased the migration potential (Fig. 3c–f) of MAECs. These results demonstrate that VEGFR2 inhibition nullifies the effects of ADSC HIF1α overexpression on MAECs in high glucose, indicating that HIF1α overexpression in ADSCs alleviates MAEC dysfunction in high glucose through VEGFR2 signaling.

### HIF1α overexpression promotes ADSC survival in high glucose/low oxygen conditions

**Fig. 4:**
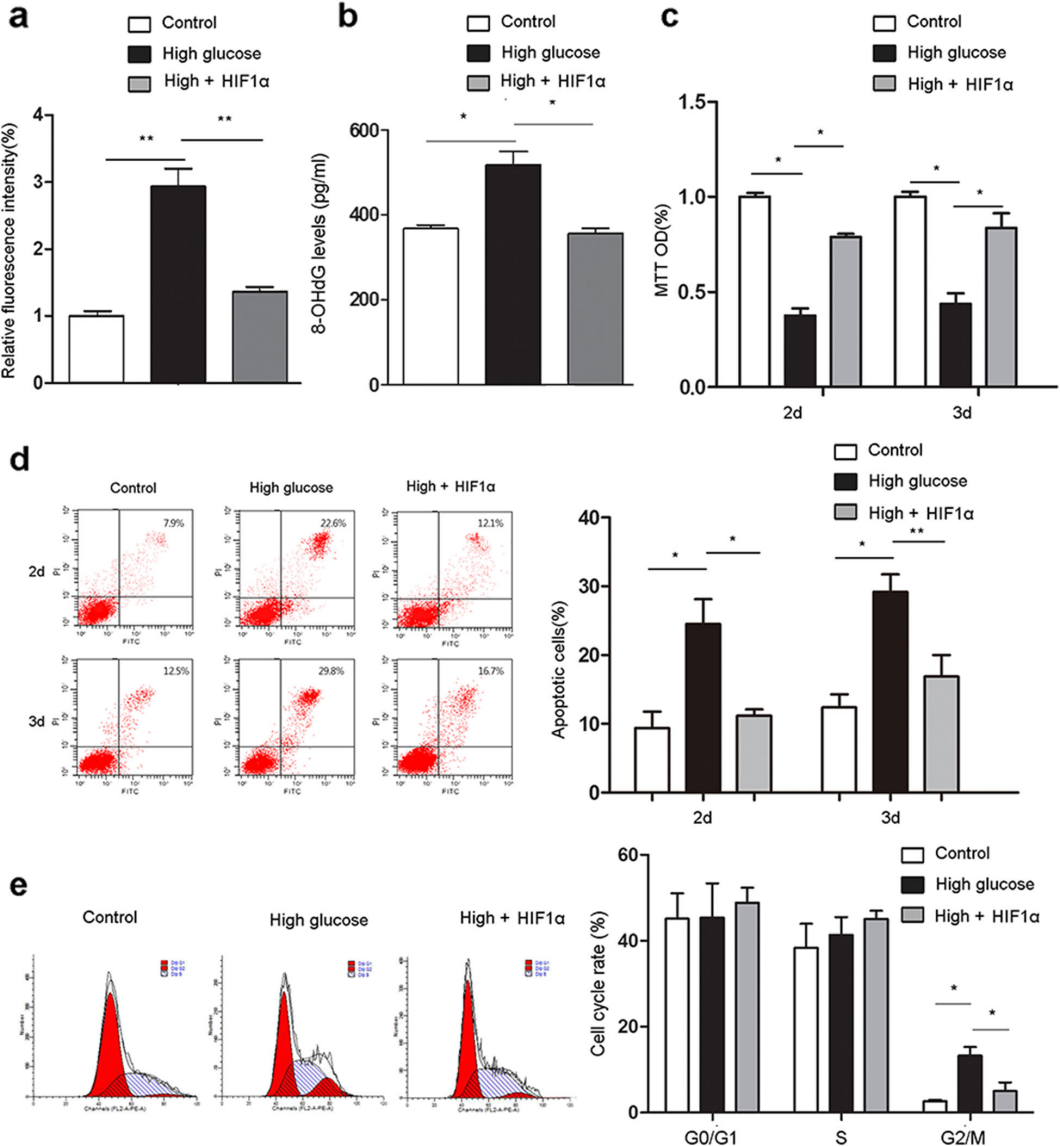
HIF1α overexpression restores ADSC survival under high glucose and low oxygen. **a** Intracellular ROS levels in control and HIF1α-overexpressing ADSCs cultured in high glucose were determined using DCFH-DA. **b** 8-OHdG levels were evaluated by ELISA. **c** The proliferation of control and HIF1α-overexpressing ADSCs under high glucose was analyzed by MTT assay. **d** Apoptosis in control and HIF1α-overexpressing ADSCs cultured in high glucose was analyzed using Annexin V-FITC/PI staining and flow cytometry. **e** Cell cycle distributions of ADSCs in each group. All experiments were performed under hypoxia. **p < 0.01; *p < 0.05; Control, normoglycemia; High, high glucose; HIF1α, HIF1α overexpression

The survival of transplanted ADSCs is the most critical factor for successful ADSC-based therapy [22, 23]. However, high glucose levels due to diabetes induce oxidative stress that damages cells and suppresses their survival [11]. Thus, we examined whether HIF1α overexpression in ADSCs could suppress ROS generation under high glucose. When cells were transduced with HIF1α prior to exposure to high glucose, HIF1α overexpression significantly reduced intracellular ROS levels (Fig. 4a). Next, 8-OHdG levels were assessed to examine the extent of DNA damage caused by oxidation. High glucose treatment significantly increased 8-OHdG levels compared with control conditions, and HIF1α overexpression significantly decreased ADSC 8-OHdG levels compared with control ADSCs (Fig. 4b). Concomitantly, HIF1α overexpression restored the proliferative potential of the ADSCs, which was suppressed by high glucose (Fig. 4c).

Next, we analyzed the effect of HIF1α overexpression on the ADSC apoptosis rate in high glucose. High glucose resulted in increased ADSC apoptosis, which was significantly reduced by HIF1α overexpression (Fig. 4d). Cell cycle analysis revealed that the percentage of cells in G2/M phase significantly increased with high glucose compared to control conditions, and HIF1α overexpression in ADSCs significantly decreased G2/M phase cells compared with high glucose (Fig. 4e); conversely, no significant changes in the G0/G1 or S phases were observed. Together, these results demonstrate that HIF1α overexpression in ADSCs may repress ROS generation, prevent DNA damage, improve cell cycle arrest, and promote ADSC proliferation and survival in diabetic environments.

### HIF1α overexpression enhances diabetic wound closure by promoting the paracrine function of ADSCs

**Fig. 5:**
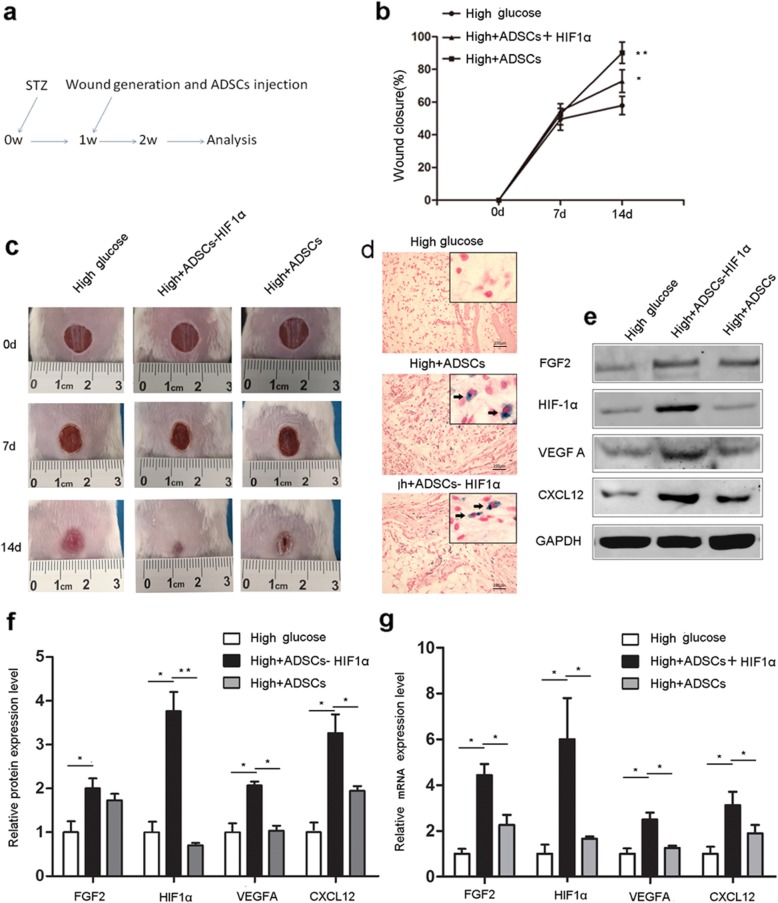
ADSC HIF1α overexpression improves diabetic wound closure and promotes angiogenic growth factor expression in mice. **a** Schematic of the in vivo experiment. **b** The rate of wound closure was quantified at the indicated times after ADSC transplantation (*n* = 12 per group). **c** Representative images of wounds 0, 7, and 14 days after ADSC transplantation. **d** Migrated ADSCs (indicated by black arrows) were stained with Prussian blue. **e** FGF2, HIF1α, VEGFA, and CXCL12 protein levels in tissues surrounding the wounds of diabetic mice transplanted with control or HIF1α-overexpressing ADSCs. **f** Quantification of FGF2, HIF1α, VEGFA, and CXCL12 protein expression levels. **g** FGF2, HIF1α, VEGFA, and CXCL12 mRNA levels from tissues surrounding the wounds of diabetic mice transplanted with control or HIF1α-overexpressing ADSCs. Expression levels are shown relative to GAPDH and expressed as the mean ± SD. **p < 0.01;*p < 0.05; High, STZ-induced mice injected with PBS; ADSCs, STZ-induced mice transplanted with ADSCs; HIF1α, STZ-induced mice transplanted with HIF1α-overexpressing ADSCs. Scale bar, 25 μm

**Fig. 6:**
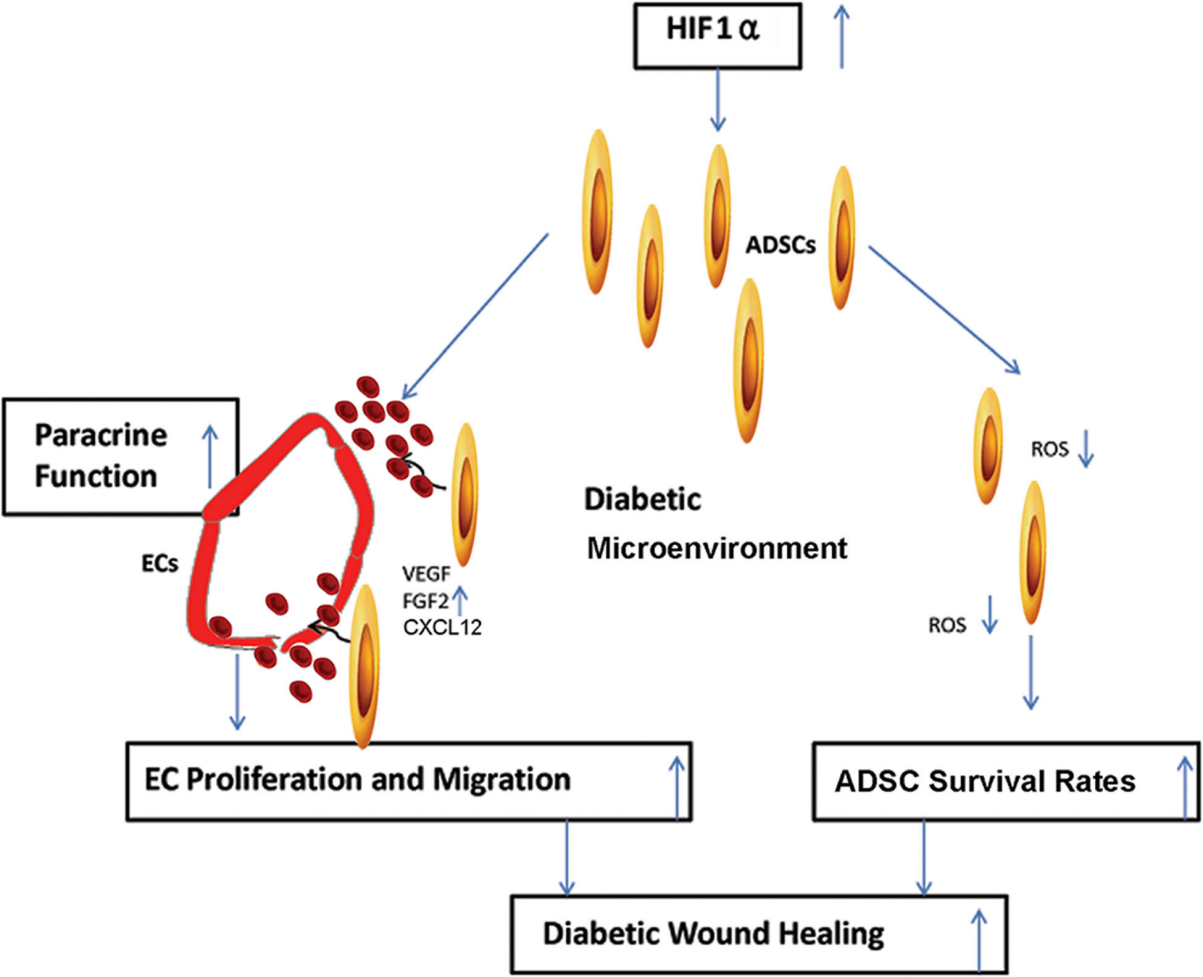
Model of the mechanisms by which HIF1α overexpression in ADSCs promotes diabetic wound healing

To assess the therapeutic effects of HIF1α-overexpressing ADSCs on diabetic wound healing, we established a mouse model of diabetic wound healing and transplanted the wounds with control or HIF1α-overexpressing ADSCs (Fig. 5a). The in vivo wound repair ability of ADSCs was examined by assessing wound closure rates. As presented in Fig. 5b and c, on the 7th day, there was a small but insignificant increase in the healing rate in the ADSC transplantation group compared to the control group. However, on the 14th day, both control and HIF1α-overexpressing ADSCs exhibited positive effects on wound closure, and HIF1α-overexpressing ADSCs significantly promoted wound closure compared to control ADSCs (Fig. 5b, c). To visualize migrated ADSCs in the wounds, they were labeled with SPIONs and sectioned tissues were stained with Prussian blue (Fig. 5d). VEGFA, FGF2, and CXCL12 protein and mRNA expression levels were upregulated at the wound sites of mice transplanted with HIF1α-overexpressing ADSCs (Fig. 5e–g). Taken together, these results demonstrate for the first time that HIF1α overexpression in ADSCs promotes their paracrine function and survival by decreasing ROS production, thereby enhancing their therapeutic effects in diabetic wound healing (Fig. 6).

## Discussion

Diabetes affects > 9% of adults worldwide [21]. People with type 2 diabetes mellitus (T2DM) have a 25-fold higher risk of limb loss than nondiabetics, mainly due to impaired wound healing. ADSC transplantation has been used in regenerative medicine as a cellular treatment for many diseases [9, 24]. However, high glucose affects the expression of various ADSC factors, resulting in systematic impairment of their physiological pathways and paracrine function and decreased survival after transplantation [22]. High glucose and advanced glycation end products severely disrupt ADSC survival, most likely due to excessive ROS production [11, 12]. Therefore, an effective method of improving stem cell paracrine function and survival in diabetic microenvironments is urgently needed.

The physiologic response to local wound hypoxia plays a critical role in determining the success of the normal healing process. HIF1α, a master regulator of oxygen homeostasis, is an important determinant of healing outcomes that contributes to all stages of wound healing [25]. HIF1 deficiency and subsequent failure to respond to hypoxic stimuli lead to chronic hypoxia, which contributes to the formation of nonhealing diabetic ulcers [26]. A recent report demonstrated the protective mechanism of HIF1α overexpression in reinfused blood cells in enhancing diabetic ischemic wound closure [27]. Although positive regulators of HIF1 are currently in clinical trials for the treatment of several human ischemic conditions, it has been reported that HIF1α stabilization in ischemic wounds leads to impaired reepithelialization and delayed wound closure, and HIF1α overexpression can contribute to fibrosis and excessive scarring [28–30]. A recent study showed that HIF1α deletion facilitated adipose stem cell-mediated repair of renal fibrosis in diabetic mice [31]. Diabetic wound healing is a complicated pathological process occurring in a complex internal environment that features high glucose, hypoxia, and oxidative stress. However, the effects of HIF1α-overexpressing ADSCs on diabetic wound healing have not been reported. We examined whether HIF1α overexpression in ADSCs can improve their paracrine function and survival in a diabetic environment. A previous report showed that VEGFA, FGF2, and CXCL12 are major components of the stem cell secretome, which is essential for angiogenesis during wound healing [9], and our results demonstrate that HIF1α-overexpressing ADSCs robustly enhance the expression of these factors, which are crucial for cell survival and wound healing in high glucose. Endothelial dysfunction is an important contributor to compromised wound healing in this context, which manifests in diabetes, atherosclerosis, fibrosis, and vascular occlusion. ECs display high sensitivity to radiation injury, yet these cells play an essential role in wound healing. ADSCs secrete various cytokines and growth factors to support the functions of other cells, including their growth and migration [25], and media derived from hypoxic preconditioned ADSCs can support EC survival and endothelial tube formation in vitro [26]. We established a coculture model to examine if HIF1α overexpression in ADSCs prevents EC dysfunction in high glucose. MAEC proliferation, survival, and migration in high glucose were significantly improved when cocultured with HIF1α-overexpressing ADSCs compared with control ADSCs. This suggests that ADSCs compensate for endothelial dysfunction in diabetic microenvironments by restoring growth factor expression.

VEGF isoforms belong to the platelet-derived growth factor family and play central roles in the regulation of angiogenesis. VEGFR2 exhibits strong tyrosine kinase activity in response to proangiogenic signals and regulates EC proliferation, migration, vascular permeability, and secretion, making it a critical factor in EC function [32]. A high glucose-induced reduction in VEGFR2 cell surface abundance in diabetic mice was reversed by treatment with the antioxidant *N*-acetyl-l-cysteine, suggesting a causative role for oxidative stress. The results of this study demonstrate that HIF1α overexpression in ADSCs restored MAEC VEGFR2 function and reduced high glucose-induced MAEC dysfunction [33].

ADSCs participate in various aspects of the wound healing process, including enhancing epidermal cell growth, angiogenesis, collagen deposition, anti-inflammatory effects, and wound closure [34, 35]. However, the therapeutic effects of ADSCs are limited by poor survival after transplantation [36]. This problem is even more severe in diabetic wound healing, as high glucose induces ROS generation, resulting in DNA damage and subsequent apoptosis [37]. Thus, increasing the antioxidant defenses of ADSCs is crucial to improve their survival and enable successful ADSC-based therapy for diabetic wound healing. This study indicates that HIF1α overexpression in ADSCs promotes cell survival under high glucose and low oxygen by stimulating the production of angiogenic growth factors, repressing ROS generation, and preventing DNA damage, resulting in accelerated diabetic wound healing.

Wound healing is a complex process that involves wound contraction, and a limitation of this study is that we did not examine the effects of wound contraction on healing in our in vivo model. In future experiments, it will be important to use splints to fix the mouse skin to prevent skin wound contraction before assaying the effects of ADSC HIF1α overexpression on wound healing.

## Conclusions

The data indicates that HIF1α overexpression in ADSCs enhances diabetic wound healing by promoting their paracrine function and preventing DNA damage caused by ROS production, increasing their survival. These beneficial effects significantly accelerated wound closure in diabetic mice transplanted with HIF1α-overexpressing ADSCs. These findings suggest that transplantation of HIF1α-overexpressing ADSCs into injury sites is a promising novel therapeutic strategy for diabetic wound healing, which could also be used in combination with other ADSC-based treatments. We propose that the transplantation of HIF1α-overexpressing ADSCs is an effective and safe alternative to proangiogenic cell transplantation in the treatment of diabetic wounds.

## Data Availability

The datasets generated and/or analyzed during the current study are included within the article and are available from the corresponding author on reasonable request.

## Abbreviations

ADSCs: Adipose-derived stem cells
DCFH-DA: 2,7-Dichlorodihydrofluorescein diacetate
EC: Endothelial cells
FGF2: Fibroblast growth factor 2
MAECs: Mouse aortic endothelial cells
PBS: Phosphate-buffered saline
8-OHdG: 8-Hydroxydeoxyguanosine
ROS: Reactive oxygen species
CXCL12: C-X-C motif chemokine ligand 12
SPIONs: Superparamagnetic polyethyleneimine-coated iron oxide nanoparticles
VEGFA: Vascular endothelial growth factor A
VEGFR2: Vascular endothelial growth factor receptor 2
